# Supplementary material for: Causal Relationship between Aging and Anorexia Nervosa: A White-Matter-Microstructure-Mediated Mendelian Randomization Analysis
Source: Biomedicines. 2024 Aug 16;12(8):1874. doi: 10.3390/biomedicines12081874 (PMC11351342; doi:10.3390/biomedicines12081874)
Supplement: Supplementary file 1 [file biomedicines-12-01874-s001.zip › Supplementary Figure/Figure S4/Singe SNP plot of MR analysis for TL to ubm-b-2053.pdf]

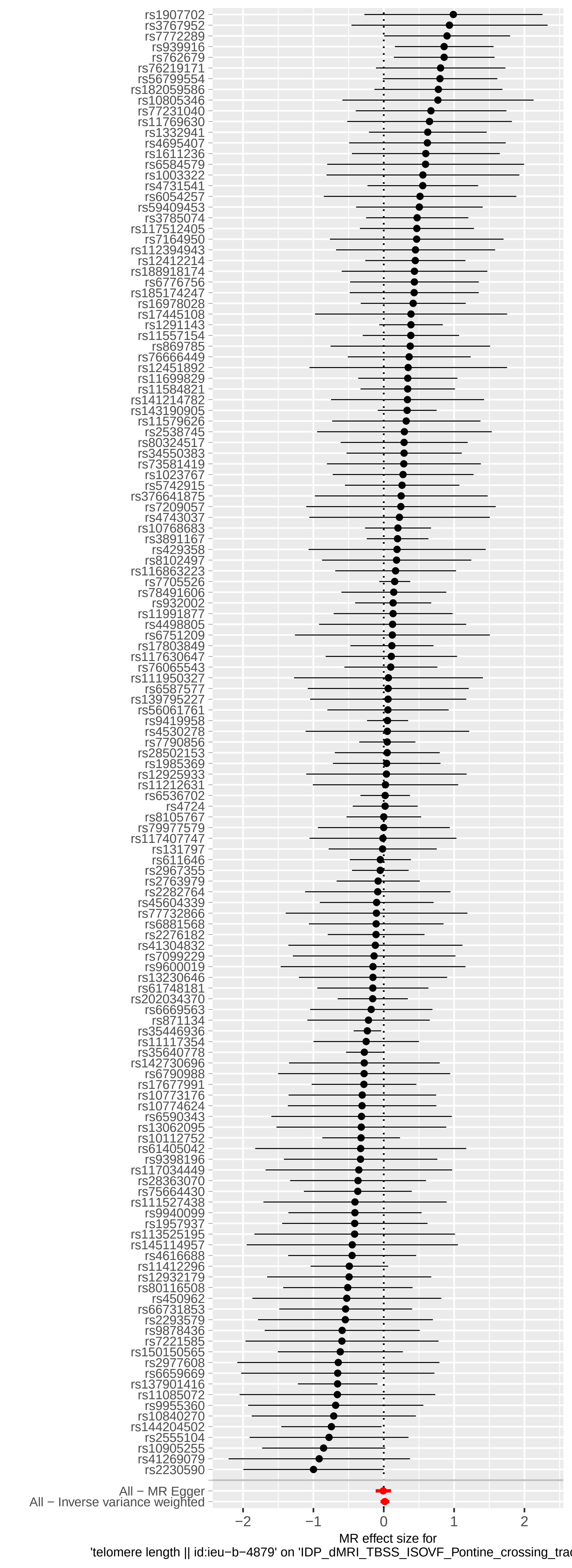
rs1907702
rs3767952
rs7772289
rs939916
rs762679
rs76219171
rs56799554
rs182059586
rs10805346
rs77231040
rs11769630
rs1332941
rs4695407
rs1611236
rs6584579
rs1003322
rs4731541
rs6054257
rs59409453
rs3785074
rs117512405
rs7164950
rs112394943
rs12412214
rs188918174
rs6776756
rs185174247
rs16978028
rs17445108
rs1291143
rs11557154
rs869785
rs76666449
rs12451892
rs11699829
rs11584821
rs141214782
rs143190905
rs11579626
rs2538745
rs80324517
rs34550383
rs73581419
rs1023767
rs5742915
rs376641875
rs7209057
rs4743037
rs10768683
rs3891167
rs429358
rs8102497
rs116863223
rs7705526
rs78491606
rs932002
rs11991877
rs4498805
rs6751209
rs17803849
rs117630647
rs76065543
rs111950327
rs6587577
rs139795227
rs56061761
rs9419958
rs4530278
rs7790856
rs28502153
rs1985369
rs12925933
rs11212631
rs6536702
rs4724
rs8105767
rs79977579
rs117407747
rs131797
rs611646
rs2967355
rs2763979
rs2282764
rs45604339
rs77732866
rs6881568
rs2276182
rs41304832
rs7099229
rs9600019
rs13230646
rs61748181
rs202034370
rs6669563
rs871134
rs35446936
rs11117354
rs35640778
rs142730696
rs6790988
rs17677991
rs10773176
rs10774624
rs6590343
rs13062095
rs10112752
rs61405042
rs9398196
rs117034449
rs28363070
rs75664430
rs111527438
rs9940099
rs1957937
rs113525195
rs145114957
rs4616688
rs11412296
rs12932179
rs80116508
rs450962
rs66731853
rs2293579
rs9878436
rs7221585
rs150150565
rs2977608
rs6659669
rs137901416
rs11085072
rs9955360
rs10840270
rs144204502
rs2555104
rs10905255
rs41269079
rs2230590
All − MR Egger
All − Inverse variance weighted
−2
−1
0
1
2
MR effect size for
'telomere length || id:ieu−b−4879' on 'IDP_dMRI_TBSS_ISOVF_Pontine_crossing_trac
